# O045: Acquisition of extended-spectrum beta-lactamase (ESBL) positive E.coli in the community: the impact of cultural background and diet

**DOI:** 10.1186/2047-2994-2-S1-O45

**Published:** 2013-06-20

**Authors:** R Leistner, E Meyer, P Gastmeier, P Dem, Y Pfeifer, C Eller, F Schwab

**Affiliations:** 1 Institute of Hygiene and Environmental Medicine, Charité Berlin, Berlin, Germany; 2 Nosocomial Infections, Robert Koch Institute, Wernigerode, Germany

## Introduction

The prevalence of ESBL producing *E.coli* strains in the community has strongly risen since recent years. Travel to high endemic countries has been identified as a risk factor for community-acquired colonization with these bacteria. Until today further factors influencing the spread of ESBL in the community have not been sufficiently analyzed.

## Objectives

The objective of this study was to assess risk factors for a community-acquired colonization with ESBL positive *E.coli*.

## Methods

From May 2011 to January 2012 we performed a case control study at the Charité university hospital Berlin. Cases were defined as patients diagnosed with ESBL positive *E.coli* colonization within 72 h after admission. Controls were patients with ESBL negative *E.coli* colonization. Cases and controls with ESBL colonization within the last 12 months were excluded. In a questionnaire based interview we assessed parameters like body mass index (BMI), nutritional habits, travel habits, recent hospital admission, recent use of antibiotics and household situation. We assessed the impact of cultural background by assessing the patients’ best mastered language. ESBL positive strains were further analyzed by PCR to determine the ESBL genotype at the Robert Koch Institute, Wernigerode. Univariable and multivariable analysis were performed to identify independent risk factors for acquisition of ESBL positive *E.coli* strains.

## Results

Within the study period we included 85 cases and 170 controls. Median age was 67 years (IQR 54-73, p=0.482) 56% of the study population was male (p=0.714). The most common ESBL genotypes were CTX-M-1 (44%, n=37) and CTX-M-15 (28%, n=24). Asian mother tongue (OR=13 4; p<0.001) and frequent pork meat consumption (>2 x per week) (OR=3.5.p<0.001) were independent risk factors for colonization with ESBL in the conditional regression analysis.

## Conclusion

Patients with cultural background of countries where ESBL is highly endemic might have a higher risk for colonization with ESBL. Furthermore the frequent consumption of certain types of meat can be associated with ESBL colonization. Common ESBL genotypes in the community are CTX-M-1 and CTX-M-15.

## Disclosure of interest

None declared.

